# Factors Contributing to Massive Blood Loss on Peripartum Hysterectomy for Abnormally Invasive Placenta: Who Bleeds More?

**DOI:** 10.1155/2016/5349063

**Published:** 2016-08-17

**Authors:** Hironori Takahashi, Akihide Ohkuchi, Rie Usui, Hirotada Suzuki, Yosuke Baba, Shigeki Matsubara

**Affiliations:** Department of Obstetrics and Gynecology, Jichi Medical University, 3311-1 Shimotsuke, Tochigi 329-0498, Japan

## Abstract

*Introduction*. To identify factors that determine blood loss during peripartum hysterectomy for abnormally invasive placenta (AIP-hysterectomy).* Methods*. We reviewed all of the medical charts of 11,919 deliveries in a single tertiary perinatal center. We examined characteristics of AIP-hysterectomy patients, with a single experienced obstetrician attending all AIP-hysterectomies and using the same technique.* Results*. AIP-hysterectomy was performed in 18 patients (0.15%: 18/11,919). Of the 18, 14 (78%) had a prior cesarean section (CS) history and the other 4 (22%) were primiparous women. Planned AIP-hysterectomy was performed in 12/18 (67%), with the remaining 6 (33%) undergoing emergent AIP-hysterectomy. Of the 6, 4 (4/6: 67%) patients were primiparous women. An intra-arterial balloon was inserted in 9/18 (50%). Women with the following three factors significantly bled less in AIP-hysterectomy than its counterpart: the employment of an intra-arterial balloon (4,448 ± 1,948 versus 8,861 ± 3,988 mL), planned hysterectomy (5,003 ± 2,057 versus 9,957 ± 4,485 mL), and prior CS (5,706 ± 2,727 versus 9,975 ± 5,532 mL). Patients with prior CS (−) bled more: this may be because these patients tended to undergo emergent surgery or attempted placental separation.* Conclusion*. Patients with intra-arterial balloon catheter insertion bled less on AIP-hysterectomy. Massive bleeding occurred in emergent AIP-hysterectomy without prior CS.

## 1. Introduction

The rate of an abnormally invasive placenta (AIP) (placenta accreta, increta, or percreta) recently increased. AIP usually requires hysterectomy. Although many previous researchers, including our team, devised various techniques for peripartum hysterectomy for AIP (AIP-hysterectomy) [1], this surgery is still challenging [2]: it usually leads to massive hemorrhage, even causing maternal death.

Various efforts to identify associations among some factors and surgical blood loss in AIP-hysterectomy have been made: their determination may promote more effective surgery. Factors tested so far were antenatal diagnosis of AIP (+) versus (−), intra-arterial temporary balloon catheter placement (intra-arterial balloon) (+) versus (−) [3], and some background factors. However, definite data are still lacking. For example, antenatal diagnosis of AIP did [4, 5], or did not, [6, 7] reduce the amount of bleeding during AIP-hysterectomy. One possible reason for this may be that, in previous studies, this surgery was performed by different obstetricians with various techniques.

What factors determine blood loss during AIP-hysterectomy? We attempted to answer this question by examining 18 consecutive cases treated in a single tertiary perinatal center, importantly, with a single experienced obstetrician (SM) attending all 18 surgeries and using the same technique.

## 2. Materials and Methods

We reviewed all the medical charts of 11,919 deliveries (singleton: 10,948, multiple: 971; January 2004–December 2014) that were managed at our center, one of the largest in Japan. We retrieved 18 AIP-hysterectomy patients, with AIP confirmed histologically. We examined the characteristics of these AIP-hysterectomy patients: age, parity, number of prior cesarean sections (CS), mode of conception, placental location, delivery week, intra-arterial balloon (+) or (−), operative time, time zone of the surgery (daytime, 9:00–17:00, or nighttime, 17:00–9:00), amount of blood loss, transfusion (red blood cells, fresh frozen plasma, or platelet concentrates), birth weight, and Apgar scores. The placental location was diagnosed just before delivery according to the criteria of the Japan Society of Obstetrics and Gynecology (http://www.jsog.or.jp/activity/pdf/gl_sanka_2014.pdf). In total placenta previa (PP), the placenta completely covers the entire internal os, with the placental margin >2 cm from the internal os; in partial PP, the placenta partially covers the internal os and the placenta margin is within <2 cm from the internal os; in marginal PP, the edge of the placenta touches the internal os, not covering it; and in low-lying placenta, the lower edge of the placenta is located within 2 cm from the internal os.

The preoperative diagnosis of AIP was made by both repeated ultrasound and magnetic resonance imaging (MRI): (1) multiple placental lacunae (>1 cm diameter); (2) absence of hypoechoic retroplacental zone; and (3) abnormalities of uterine serosa-bladder interface (its bulging into bladder and hypervascularity) when the placenta was present beneath the bladder [8, 9]. Experienced radiologists interpreted the MRI findings. Our protocol for AIP was primary hysterectomy; we do not employ a conserving strategy with the placenta remaining* in situ*. For patients in whom we did not suspect AIP, cord traction and manual removal of the placenta were performed similarly in CS without AIP.

The technical details of AIP-hysterectomy were previously described [1]: we fundamentally used this technique. In short, on planned AIP-hysterectomy, the intra-arterial balloon was inserted before the surgery. The balloon was placed in the common iliac artery except for 2 patients in whom it was placed in the internal iliac artery [10]. The balloon was inflated at the time of massive bleeding based on the operator's judgment. The balloon occlusion time was up to 40 minutes. General anesthesia was applied. Planned hysterectomy was performed during the late preterm period, as previously recommended [4, 11]. A multidisciplinary team approach was employed, with radiologists, urologists, anesthesiologists, and neonatologists attending. Supracervical hysterectomy was performed in some cases based on the attending surgeons' judgment. We did not perform internal iliac artery ligation.

When we did not suspect the presence of AIP in patients with PP, elective CS was scheduled at 37 gestational weeks. If the placenta occupied the anterior wall, we determined the incision line by transuterine ultrasound to avoid a transplacental approach. If significant bleeding occurred after placental removal, we employed uterine compression suture [12–14], an intrauterine hemostatic balloon [15, 16], and holding the cervix [17, 18]. The amount of blood loss included intraoperative hemorrhage and the bleeding within <24 hours postpartum, that is, early postpartum bleeding.

CS (without PP or AIP) was usually performed by an attending physician and residents. CS for PP and/or elective AIP-hysterectomy was performed by experienced obstetricians (chief, associate, or assistant professor) with an attending physician and residents. A chief professor (SM) and a specialist of AIP-hysterectomy, attended, or at least supervised, all 18 AIP-hysterectomies. When antenatal AIP diagnosis (+) patients bled before planned surgery or antenatal AIP diagnosis (−) patients proved to have AIP during surgery; emergent AIP-hysterectomy was performed, in which a chief professor also participated: in the daytime, his participation was required for several minutes whereas in the nighttime, it was required for 30 minutes. An intra-arterial balloon was not employed during the nighttime. Transfusion was judged by the attending obstetricians or anesthesiologists. A cell saver was not used. Autologous blood donation (before surgery) was performed up to 800 mL in patients with PP, when the maternal hemoglobin level was >10 g/dL.

This study was approved by the Ethics Committee of our institute. We compared variables between the AIP-hysterectomy (*n* = 18) and control groups: the latter consisted of all patients without AIP-hysterectomy (*n* = 11,901). The *t*-test (two-tailed) and Fisher's exact test were used to compare maternal characteristics and obstetric and neonatal outcomes between the groups. All analyses were performed using JMP version 10 (SAS Institute, Tokyo, Japan), with *P* < 0.05 considered as significant.

## 3. Results

AIP-hysterectomy was performed in 18 patients (0.15%: 18/11,919). Patients with AIP-hysterectomy were older, delivered earlier, bled more, and gave birth to lighter infants than the controls, with all being significant (Table 1).

Table 2 shows the clinical backgrounds and characteristics of patients with AIP-hysterectomy. The mode of delivery was CS in all 18 patients. Of the 18, 14 (78%) had a prior CS history and the other 4 (22%) were primiparous women. Three (17%) underwent artificial reproductive technology (ART). One patient (Case 18) had dichorionic-diamniotic twins: [19] one cotwin's placenta was in the uterine body and the other cotwin's placenta had marginal previa, with the former, interestingly, having accreta. PP was present in 17/19 placentas (90%: Case 18 was a twin pregnancy and, thus, total placentas numbered 19). The remaining 2 placentas (2/19: 10%) had a normal location and were not PP, of which one (Case 18) was of a cotwin placenta, as described above. Planned AIP-hysterectomy was performed in 12/18 (67%), with the remaining 6 (33%) undergoing emergent AIP-hysterectomy. Of 6 patients, 4 (4/6: 67%) were primiparous women. Histological examination confirmed placenta accreta (*n* = 9), increta (*n* = 4), or percreta (*n* = 5). An intra-arterial balloon was inserted in 9/18 (50%). Bleeding after partial placental separation (manually or spontaneously) during surgery occurred in 11/18 (61%). The average time required for the surgery was 205 ± 78.3 (range: 119–356) min and the average blood loss was 6,655 ± 3,798 (range: 1,620–18,000) mL.

Table 3 shows that patients with the following three factors significantly bled less in AIP-hysterectomy than its counterpart: the employment of an intra-arterial balloon (4,448 ± 1,948 versus 8,861 ± 3,988 mL), planned hysterectomy (5,003 ± 2,057 versus 9,957 ± 4,485 mL), and prior CS (5,706 ± 2,727 versus 9,975 ± 5,532 mL) (*t*-test).

Table 4 shows the relationship between the procedure and blood loss in patients with emergent AIP-hysterectomy. In these cases, we could not confirm the presence of AIP before surgery and, thus, we removed the placenta manually, causing massive bleeding. In 4 primiparous women, we first tried to conserve the uterus by various hemostatic procedures, without success. Hysterectomy was performed after these efforts. The amount of bleeding (blood loss until manual removal of the placenta + that from placental removal to the start of hysterectomy) reached an average of 4,768 mL at the time of starting hysterectomy.

All the mothers and neonates had a good outcome (data not shown). Six patients had surgical complications: bladder injury (*n* = 4: 3 increta (Cases 2, 8, and 17) and 1 percreta (Case 4)) and intestinal injury necessitating intestinal resection (*n* = 2: 1 percreta (Case 13) and 1 accreta (Case 18)) (Table 2). All 6 recovered completely without sequelae. No complications associated with the intra-arterial balloon were observed.

## 4. Discussion

Patients with intra-arterial balloon insertion bled less than those without it. Patients with planned AIP-hysterectomy bled less than those with emergent surgery. Patients with a prior CS history bled less than their counterparts. In short, patients with intra-arterial balloon (+), planned hysterectomy (+), or prior CS (+), bled less.

Patients with an intra-arterial balloon bled less than those without it. Previous studies showed controversial results regarding this [20–27]. Of those, a well-designed retrospective case-control study showed that intra-arterial balloon use significantly reduced blood loss [23]: it reduced blood loss by more than 2,500 mL and the rate of massive transfusion by approximately half (27 versus 55%; 31 versus 52%, resp.). Recently, another group also reported the efficacy of intra-arterial balloon using historical control [24]. Another retrospective study showed that intra-arterial balloon use significantly reduced blood loss in patients with placenta “percreta,” although it did not reduce that in patients with placenta “accreta/increta” [25]. Some others showed that balloon use did not reduce the amount of bleeding. However, these studies had marked limitations: one study had entry number differences (balloon *n* = 6, control *n* = 22) [20], and another had the potential selection bias of differences in the number of prior CS [21]. Another recent study showed that intra-arterial balloon use did not reduce blood loss: blood loss and the number of transfused red blood cells were the same between intra-arterial balloon and control groups [27]. Although their study number was small (balloon *n* = 13 versus control *n* = 14), this was the first randomized controlled trial regarding this issue. However, there are also concerns in this study. First, approximately half had a conserved uterus, and, thus, histological evidence of AIP was not obtained. Second, the average blood loss was approximately 1,600 mL, being a relatively small amount, even in control groups. Previous reports, similar to our present study, showed average blood loss of 3,000–5,500 mL in AIP [28], suggesting that the patients may have suffered “less severe” AIP. Third, internal-iliac-artery occlusion was employed in this study, which may not have achieved satisfactory hemostasis. Common-iliac-artery-occlusion may more effectively reduce blood loss than internal-iliac-artery occlusion [10, 29]. Taking all these into account, this study may not show “real-world” data.

Ischemic adverse events associated with intra-arterial balloon, especially artery thromboses (popliteal artery [24, 30], iliac artery [21, 24, 31], and femoral artery [21]), have been reported. The incidence rate of these adverse events was reported to be approximately 15% [21, 24]. Whereas some resolved with expectant management [21, 24], others required additional interventions [21, 30]. Many factors (occlusion time, occlusion pressure, patients' arterial condition and thrombotic tendency, etc.) may be involved in the pathogenesis of this adverse event: no definite methods to prevent these adverse events have been demonstrated. Strategies to prevent the ischemic events and, if they occurred, strategies to detect them early should be established. At present, caution should be made for this adverse event.

Patients with planned hysterectomy bled less than those with emergent hysterectomy. This is consistent with the previous studies [32, 33]. In planned surgery, hysterectomy can be performed with a multidisciplinary team in an elective manner, with the placenta remaining undelivered. On the contrary, emergent hysterectomy was performed due to (1) acute massive bleeding before the planned date of surgery in patients with an antenatal diagnosis of AIP or (2) unexpectedly “encountering” AIP in patients without an antenatal diagnosis of AIP. In both cases, a multidisciplinary team is unavailable, and in the latter case, the placenta sometimes may be removed manually with resultant massive bleeding.

We demonstrated two findings contradicting previous beliefs: the beliefs are that AIP patients with prior CS (+) or PP (+) may bleed more than prior CS (−) or PP (−) patients. To our knowledge, no evidence has been reported regarding the relationship between these two factors and amount of bleeding at AIP-hysterectomy; however, since these two are well known as risk factors of AIP [34], obstetricians may consider that these two, CS and PP, may also cause massive bleeding on AIP-hysterectomy. The present study showed contradictory data: (1) patients without prior CS bled more than their counterparts, and (2) some patients with AIP in the normal placental position (not PP) had massive bleeding (two patients: 5,550 mL (Case 13) and 18,000 mL (Case 18)): prior CS (−) or PP (−) patients bled more. This may well reflect our clinical impression: we did not suspect AIP in prior CS (−) or PP (−) patients before or during surgery and, thus, we manually removed the placenta as usually. After the placental separation, we noticed the presence of AIP and, at first, we employed various hemostatic procedures such as uterine compression suture or intrauterine balloon use, without achieving hemostasis. During these procedures, bleeding continued and we finally decided on hysterectomy. Notably, 4 of 6 patients who required emergent hysterectomy were primiparous women. In retrospect, we should have resorted to hysterectomy just following manual placental separation. This may indicate that physicians must not be any less cautious regarding massive bleeding on AIP-hysterectomy even in patients without prior CS, without PP, or primiparous women.

In this study, there were no differences in blood loss between nighttime versus daytime surgery (9,071 ± 5,125 versus 5,725 ± 2,882 mL, *P* = 0.095). Several studies showed that the use of a multidisciplinary team improved the outcome of AIP-hysterectomy [35, 36]. A multidisciplinary team was fundamentally unavailable at night in our institute. This may be because we planned elective surgery in the preterm period for very severe cases and, thus, less severe cases may have belonged to the “nighttime” surgery group.

The present study population is not sufficient enough to demonstrate definite statistical evidence. Considering the rarity of this surgery, this may be a limitation of a study based on single-center experience. Furthermore, employment or unemployment of intra-arterial balloon, for example, was chosen on the doctors' judgment and not randomly assigned. This may be limitation general to a retrograde observational study. Our strengths were as follows: (1) during the study period, we employed the same procedure for AIP-hysterectomy [1], (2) the surgical team members were generally the same, and (3) an obstetric surgery specialist, especially a specialist of AIP-hysterectomy (SM), attended all these surgeries. Many institutes worldwide may not have 24-hour 365-day practice: team rotation is impossible. For a difficult surgery, a specialist(s) for the corresponding surgery may be called. The present situation may represent those of many institutes worldwide, and, thus, the present data may reveal “real-world experience.”

In conclusion, patients with intra-arterial balloon catheter insertion bled less on AIP-hysterectomy. Massive bleeding occurred in emergent AIP-hysterectomy without prior CS or PP. Although this study was retrospective with relatively small number of cases, we believe that the results reflect “real-world experience”: describing this experience may contribute to better outcome of this challenging surgery.

## Figures and Tables

**Table 1:** Patients' backgrounds of AIP-hysterectomy group versus control group.

	Hysterectomy for AIP (*n* = 18)	Control (*n* = 11,901)	*P* value
Age (years)	36.8 ± 4.7	31.9 ± 5.3	<0.001
Multiple pregnancy	1 (5.6%)	970 (8.2%)	1.000
GA at delivery (week)	35.1 ± 2.2	38.0 ± 2.9	<0.001
Blood loss (mL)	6,655 ± 3,798	635 ± 561	<0.001
Birth weight (g)	2,261 ± 452	2,707 ± 644	<0.001
CS	18 (100%)	5,768 (48.5%)	<0.001

AIP: abnormally invasive placenta, CS: cesarean section, and GA: gestational age.

The *t*-test and Fisher's exact test are applied.

**Table 2:** Details of patients with AIP who underwent hysterectomy.

Case	Age	G	P	Number of prior CS	Mode of conception	Placental location	Degree of PP	Pathology	Delivery week	Hysterectomy	Placental removal	Bleeding after partial placental separation^*∗*^	Intra-arterial balloon	Operative time (min)	Time zone of surgery	Blood loss (mL)	Birth weight (g)	Apgar 1, 5 min	Surgical complication
1	38	3	1	1	Natural	PP, anterior	Total	Percreta	34–3	Planned	−	−	+	373	Day	6,850	2988	3, 5	
2	31	3	1	1	Natural	PP, anterior	Total	Increta	34–5	Planned	−	−	+	219	Day	6,656	2134	2, 2	Bladder injury
3	36	2	2	2	Natural	PP, anterior	Total	Percreta	36–3	Planned	−	−	+	123	Day	3,130	2530	5, 7	
4	35	3	2	2	Natural	PP, anterior	Total	Percreta	30–1	Planned	−	−	+	226	Day	6,700	1612	1, 3	Bladder injury
5	43	7	7	2	Natural	PP, anterior	Total	Accreta	35–1	Planned	−	+	+	184	Day	4,720	2360	2, 3	
6	38	2	2	2	Natural	PP, posterior	Total	Accreta	36–4	Planned	−	+	+	143	Day	4,440	3152	5, 8	
7	30	4	2	2	Natural	PP, anterior	Total	Accreta	34–0	Planned	−	−	+	190	Day	3,420	2018	3, 4	
8	38	3	3	3	Natural	PP, anterior	Total	Increta	31–2	Planned	−	−	+	134	Day	1,620	1322	3, 6	Bladder injury
9	41	4	3	3	Natural	PP, anterior	Total	Percreta	34–5	Planned	−	−	−	180	Night	8,500	2392	4, 6	
10	43	1	1	1	Natural	PP, anterior	Total	Increta	35–3	Planned	−	+	−	150	Night	5,625	2162	1, 8	
11	32	1	1	1	Natural	PP, anterior	Total	Accreta	33–6	Planned	−	+	−	156	Night	5,880	2222	2, 6	
12	31	3	1	1	Natural	PP, anterior	Total	Accreta	34–5	Planned	−	+	+	119	Day	2,500	2336	8, 9	
13	38	1	0	0	ART	Normal, posterior	Not PP	Percreta	37–5	Emergent	+	+	−	320	Day	5,550	2322	8, 9	Intestinal injury
14	38	1	1	1	Natural	PP, posterior	Low-lying	Accreta	38–5	Emergent	+	+	−	175	Day	7,830	2318	8, 9	
15	32	2	1	1	Natural	PP, anterior	Total	Accreta	36–1	Emergent	+	+	−	162	Day	12,010	2416	8, 9	
16	35	0	0	0	Natural	PP, anterior	Total	Accreta	37–1	Emergent	+	+	−	193	Day	9,000	2270	5, 6	
17	40	0	0	0	ART	PP, posterior	Partial	Increta	37–2	Emergent	+	+	−	287	Night	7,350	2848	8, 9	Bladder injury
18	45	0	0	0	ART	PP, posterior, Normal, posterior	Marginal, not PP	Accreta	33–3	Emergent	+	+	−	356	Night	18,000	1708, 1840	8,9/8,9	Intestinal injury

AIP: abnormally invasive placenta, ART: assisted reproductive technology, CS: cesarean section, PP: placenta previa, G: gravida, and P: parity.

^*∗*^Partial placental separation occurred during the surgery, which caused bleeding.

**Table 3:** Blood loss according to intra-arterial balloon placement, planned surgery, or prior CS.

		Blood loss (mL, mean ± SD)	*P* value
Intra-arterial balloon	Inserted (*n* = 9)/not inserted (*n* = 9)	4,448 ± 1,948/8,861 ± 3,988	0.009
Hysterectomy	Planned (*n* = 12)/emergent (*n* = 6)	5,003 ± 2,057/9,957 ± 4,485	0.005
Prior CS	Present (*n* = 14)/absent (*n* = 4)	5,706 ± 2,727/9,975 ± 5,532	0.043

CS: cesarean section.

**Table 4:** Process of blood loss in patients with emergent AIP-hysterectomy.

Case	Para	History of CS	Obstetric complication	Total blood loss (mL)	Blood loss until manual placental removal (mL)	Decision time for hysterectomy from start of CS (min)	Hemostatic procedure	Blood loss from placental removal to start of hysterectomy (mL)	Blood loss during hysterectomy (mL)
13	1	Present	Leiomyoma	5,550	1,295	26	Manual compression, gauze packing	920	3,335
14	1	Present	Leiomyoma	7,830	3,205	35	Manual compression, gauze packing	827	3,798
15	0	Absent		12,010	2,790	32	HC	2,100	7,120
16	0	Absent		9,000	1,925	399	HC, IUB, and M-Y	4,825	2,250
17	0	Absent	Leiomyoma	7,350	1,365	809	HC	305^*∗*^	5,680
18	0	Absent	Twin pregnancy	18,000	2,500	729	HC, IUB	6,550	8,950

AIP: abnormally invasive placenta, CS: cesarean section, HC: holding the cervix, IUB: intrauterine balloon, and M-Y: Matsubara-Yano suture [12, 14]. ^*∗*^Bleeding escaped into peritoneal cavity with little vaginal bleeding. In this case, transabdominal ultrasound revealed marked fluid collection in Morrison fossa, indicating the intraabdominal bleeding, which was confirmed later, and, thus, “305 mL” indicates “measurable vaginal bleeding.”

## Abbreviations

AIP: Abnormally invasive placenta
ART: Assisted reproductive technology
CS: Cesarean section
GA: Gestational age
HC: Holding the cervix
IUB: Intrauterine balloon
MRI: Magnetic resonance imaging
M-Y: Matsubara-Yano suture
PP: Placenta previa.
